# Nonalcoholic Fatty Liver Disease and Non-Alcoholic Steatohepatitis: Current Issues and Future Perspectives in Preclinical and Clinical Research

**DOI:** 10.3390/ijms21249646

**Published:** 2020-12-17

**Authors:** Clarissa Berardo, Laura Giuseppina Di Pasqua, Marta Cagna, Plinio Richelmi, Mariapia Vairetti, Andrea Ferrigno

**Affiliations:** Unit of Cellular and Molecular Pharmacology and Toxicology, Department of Internal Medicine and Therapeutics, University of Pavia, 27100 Pavia, Italy; clarissa.berardo01@universitadipavia.it (C.B.); marta.cagna02@universitadipavia.it (M.C.); plinio.richelmi@unipv.it (P.R.); mariapia.vairetti@unipv.it (M.V.)

**Keywords:** non-alcoholic fatty liver disease, metabolic syndrome, steatohepatitis, hepatocellular carcinoma, steatosis

## Abstract

Nonalcoholic fatty liver disease (NAFLD) is a continuum of liver abnormalities often starting as simple steatosis and to potentially progress into nonalcoholic steatohepatitis (NASH), fibrosis, cirrhosis and hepatocellular carcinoma. Because of its increasing prevalence, NAFLD is becoming a major public health concern, in parallel with a worldwide increase in the recurrence rate of diabetes and metabolic syndrome. It has been estimated that NASH cirrhosis may surpass viral hepatitis C and become the leading indication for liver transplantation in the next decades. The broadening of the knowledge about NASH pathogenesis and progression is of pivotal importance for the discovery of new targeted and more effective therapies; aim of this review is to offer a comprehensive and updated overview on NAFLD and NASH pathogenesis, the most recommended treatments, drugs under development and new drug targets. The most relevant in vitro and in vivo models of NAFLD and NASH will be also reviewed, as well as the main molecular pathways involved in NAFLD and NASH development.

## 1. Introduction

In the last decades, a rise in the diffusion of chronic liver pathologies has been observed; among them, one of the most insidious is nonalcoholic fatty liver disease (NAFLD) [1]. NAFLD is characterized by the intracellular deposition of lipids in hepatocytes, often associated with a wide spectrum of metabolic abnormalities, such as dyslipidemia, hypertension, insulin resistance and diabetes; these features are collectively known as the manifestation of metabolic syndrome [2]. Under the umbrella term of “NAFLD”, adopted in 1986 [3], a wide range of pathological conditions are comprised: simple steatosis (fat accumulation in the hepatic parenchyma), non-alcoholic steatohepatitis (NASH), fibrosis, cirrhosis and hepatocellular carcinoma. In a three-year period, 20–30% of patients with simple steatosis have been found to progress toward NASH, a more severe condition in which fat accumulation is accompanied by inflammation and oxidative stress [4]. The resulting chronic inflammatory state may develop into fibrosis, cirrhosis and hepatocellular carcinoma. Patients suffering from simple steatosis present higher life expectancy than those affected by NASH, who also incur in cardiovascular damage [2]. Recent studies have demonstrated that NASH is one of the most important causes of liver transplantation in the USA, and it will become the leading cause for the request of liver donors in the next decades [5].

Recently, the name used to define NAFLD has been questioned. On one hand, the use of the term “non” has been judged to diminish the importance of the condition; on the other hand, “non-alcoholic fatty liver disease” fits the definition of “non-communicable diseases” estimated to cause >70% of global death [6]. In this context, a panel of experts recommended renaming NAFLD into metabolic (dysfunction)-associated fatty liver disease (MAFLD) [7], concomitantly with the adoption of new diagnostic criteria [8]. At the moment, a general consensus has not been achieved as some reason for criticism has been found in the proposed diagnostic criteria [9]; even though the old negative definition is generally considered outdated, over-emphasizing the metabolic dysfunctions in MAFLD might lead to the underestimation of the impact of steatosis itself in a significant group of patients at risk of disease progression [10,11].

In this review, we will discuss NAFLD pathogenesis, its progression to NASH, the currently recommended treatments, new drug candidates under development and potential new drug targets. The most relevant in vitro and in vivo models of NAFLD and NASH will also be reviewed, as well as the main molecular pathways involved in NAFLD and NASH development.

## 2. Methods

A search through PubMed was performed to identify relevant articles published until 18 November 2020. Search terms included steatosis and nonalcoholic steatohepatitis in combination with epidemiology, management, pathogenesis, fibrosis, cirrhosis, liver transplantation, hepatocellular carcinoma, experimental models, new drug targets. Additional relevant articles were identified from citations referenced in other articles, if they did not appear in the original search.

## 3. Epidemiology

It has been recently estimated that NAFLD affects 24% of the world’s population. The highest prevalence rate has been registered in South America (31%), followed by Middle East (32%), Asia (27%), USA (24%) and Europe (23%). The lowest rate, instead, was observed in Africa (14%). Based on studies conducted from 2005 to 2015, the prevalence of NAFLD is increasing and a similar trend was observed also for NASH [1]. In agreement with its metabolic origin, 42% of NAFLD subjects show metabolic syndrome (MetS) [1,12]. Metabolic syndrome (MetS) is defined as a cluster of metabolic disorders including abdominal obesity, hypertension, dyslipidemia and impaired glycemia [13]. MetS is highly prevalent worldwide, as well as its related metabolic disorders [14]. The prevalence of NAFLD was found to be significantly greater (43.2%) in patients with MetS when compared with its prevalence in the general population. In addition, the prevalence of advanced hepatic fibrosis, which was found to be 6.6% in people with mild to severe steatosis, rose to 30.3% in people with five MetS abnormalities [15]. In obese people, NAFLD recurrence ranges from 60 to 95% [12]; in a cohort study in which subjects were observed over a period of approximately 4 years, visceral adipose tissue, and not subcutaneous adipose tissue, was associated with higher risk of NAFLD with an adjusted hazard ratio of 2.23 (95% CI 1.28–3.89) [16]. The association between type-2 diabetes (T2D) and NAFLD has recently been investigated in two meta-analyses. The first one involved 35,599 T2D patients from twenty-four studies, 20,264 of them were diagnosed with NAFLD; the pooled prevalence of NAFLD in T2D patients was 59.67% (95% CI 54.31–64.92), which rose to 77.87% (95% CI 65.51–88.14) in those with obesity [17]. In another meta-analysis, obtained combining data from 80 studies (49,419 individuals), a global NAFLD prevalence of 55.5% (95% CI 47.3–63.7) was found among patients with T2D; in Europe, a prevalence of 68% (95% CI 62.1–73.0) was found, followed by West Asia (67.29%; 95% CI 60.39–73.61%), South Asia (57.87%; 95% CI 52.87–62.68%), Latin America (56.83%; 95% CI 34.05–76.98%), East Asia (52.04%; 95% CI 45.37–58.55%), United States (51.77%; 95% CI 31.33–71.64%) and Africa (30.39%; 95% CI 11.64–67.09%) [18].

In addition to the metabolic risk factors, sex and age also have a major influence on the risk of NAFLD [19]. With regard to the influence of sex, it has been observed that inconsistent findings were previously found; however, the majority of the studies did not clearly distinguish pre- and post-menopausal women, resulting in contradictory results. In more recent studies, men were found to be associated with a higher risk for advanced fibrosis in comparison with pre-menopausal women. Differently, both sexes display similar severity of fibrosis when women after menopause were considered, suggesting that estrogen may protect from advanced liver injury (reviewed by Lonardo et al., (2020)) [20]. In addition, in a study on obese individuals approximately 41 years old, NASH has been independently associated with the male sex [21]. In older patients NAFLD is more prevalent; in fact, the majority of patients were diagnosed between 40 to 50 years of age. Moreover, older age is associated with a higher risk of NASH and fibrosis [22]. However, it should be noted that, as a result of a constant increase in obesity rate, NAFLD is currently the most common cause of liver disease in children, ranging from steatosis, to NASH and fibrosis [23].

Studies based on liver biopsies have shown that patients with NASH are at the highest risk for cirrhosis. In a study, after 12 years of follow-up, risk of liver-related mortality was 6 times higher in patients with NASH than in non-NASH NAFLD patients [24]. Similar conclusions have been obtained in multiple other studies [25,26,27]. Long-term outcome of patients with NAFLD diagnosed by non-invasive techniques also provided evidences that T2D and MetS may accelerate disease progression and increase the risk of liver-related mortality [28,29,30]. Advanced fibrosis was found to induce a 2- or 3-fold increase in the risk for cardiovascular mortality in NAFLD patients, supposedly because of a proinflammatory environment and endothelial dysfunction observed in patients with NAFLD [31]. NAFLD and NASH are also well-known risk factors for developing HCC [19]. Patients with NAFLD-related fibrosis (stages 3 and 4) have approximately a 7 times higher risk for developing HCC; however, it is interesting to note that also NAFLD patients with no evidence of fibrosis may develop HCC [32]; in fact, NASH-related HCC is increasingly being seen in clinical setting, as indirectly demonstrated by the increasing number of patients being listed for liver transplantation related to HCC [19].

The variable course of NAFLD progression poses a clinical challenge that needs to be addressed by better characterizing the multiple modifiers of the disease, including the role of genetic polymorphisms, family and personal history, alcohol or drug consumption, diet, physical activity. Various topics have been proposed as relevant research priorities [33], such as the role of sex, depression and anxiety, endocrine disorders, chronic obstructive pulmonary disease and sleep apnoea syndrome in the pathogenesis of NAFLD, as well as the therapeutic role of intermittent fasting and anticoagulation [33].

## 4. Diagnosis

Considering the high prevalence of this pathology, its severe stages of progression and the difficulty encountered by the clinicians in identifying it, the development of reliable, non-invasive diagnostic techniques, able to recognize the disease in both its early and advanced stages, possibly avoiding liver biopsy, is very pressing [34].

NAFLD is often asymptomatic; in fact, patients usually discover to be affected by NAFLD only incidentally, during routine laboratory examination, when the hepatic panel reveals increased transaminase serum levels. In addition, about 80% of patients show normal ALT levels that also tend to decrease in the pathology progression from the stage of fibrosis to cirrhosis, making it unhelpful in the diagnosis and causing errors in the evaluation of clinicians, who may neglect patients potentially at risk [35].

The joint European Association for the Study of the Liver, European Association for the Study of Diabetes and the European Association for the Study of Obesity (EASL-EASD-EASO) guidelines for the management of nonalcoholic fatty liver disease recommend that simple steatosis should be diagnosed by imaging, in particular ultrasonography (US), because it is widely used and less expensive than the gold standard, magnetic resonance imaging (MRI). US, MRI or computed tomography (CT) were considered low-performance techniques for the detection of mild steatosis; however, some semi-quantitative scores (i.e., Hamaguchi score, US FLI and hepatorenal steatosis index) have been proposed to improve US performance (reviewed by Ferraioli et al., 2019) [36]. These techniques are currently considered first-line diagnostic tests in case of moderate and severe steatosis, providing also information about the hepatobiliary system. Nevertheless, the serum biomarkers investigation is the method of choice when a large scale screening is needed because the costs of imaging techniques are not sustainable [37].

Since liver enzymes and imaging are not able to predict the onset and presence of NASH [34], novel serum markers have been taken into account, such as the intermediate filament protein cytokeratin 18 (CK18), a fragment obtained after caspase-3-mediated cleavage of different substrates during the apoptotic process, one of the main mechanisms induced during NASH progression. CK18 is detected by ELISA tests in patients suffering from NASH and it is significantly higher with respect to NAFLD patients [38,39]. Unfortunately, CK18 does not provide further information than the ALT transaminase, thus the gold standard diagnostic method to discriminate NAFLD from NASH is still the liver biopsy [2,37].

In the near future, innovative diagnostic methods currently under investigation might be employed to discriminate NASH from simple steatosis; for example, recent studies by Croce et al. have shown in animal models of NAFLD-NASH that liver tissue autofluorescence (AF) could be relevant for both experimental hepatology and clinical early diagnosis. By evaluating AF spectra of fatty acids, lipofuscin-like lipopigments (indices of oxidation), NAD(P)H and flavins (indices of energy and redox state), the author were able to discriminate steatosis from advanced NASH or fibrosis in methionine choline-deficient (MCD) and Zucker rat models [40]. The same authors also demonstrated that the spectrofluorometric analysis using a fiber-optic probe is an effective method for the in situ discrimination of fatty livers suitable for transplantation from those who would be too susceptible to cold ischemia/reperfusion injury, by assessing the differences in free fatty acids relative concentration [41]. In this work, livers underwent two different kinds of preservation techniques: cold storage and machine perfusion, followed by an ex-vivo model of liver reperfusion [42], suggesting that AF could be a suitable diagnostic method not only for applied research, but also in a clinical setting [41].

Different studies have pointed to asymmetric dimethylarginine (ADMA) as a potential serum marker for NAFLD diagnosis. ADMA is a physiological compound naturally produced during protein methylation; it acts as an unspecific, competitive inhibitor of nitric oxide synthases (NOS), and its levels are often seen to be increased in patients suffering from liver disorders [43], including NAFLD and NASH [44]. An excess of circulating ADMA inhibits nitric oxide (NO) production causing vasoconstriction, an increase in platelet aggregation, endothelium cell adhesion and vascular muscle cell proliferation [45]; so, changes in ADMA levels in NAFLD/NASH patients may induce further cardiovascular complications, the major cause of death in NAFLD patients, worsened by insulin resistance, hepatic dysfunction and chronic inflammation [46]. Moreover, changes in ADMA-NOS pathway play a pivotal role in the onset and progression of NAFLD and NASH in MCD diet-fed rats [47]. However, further clinical investigations are necessary to clarify whether ADMA is a good marker for the early diagnosis of NAFLD/NASH.

More recently, it has been found that large quantities of microparticles (MPs) are released by stressed/damaged hepatocytes, contributing to the occurrence of inflammation, fibrogenesis, and angiogenesis; consequently, MPs are considered another promising biomarker for NASH [48]. By evaluating MPs, it is possible to trace the cells that originated them, so they could be considered non-invasive biomarkers for NASH. In a murine model of NAFLD, blood MV levels were seen to be increasing in a time-dependent manner, becoming significantly higher after 8-week choline-deficient diet administration, a time point when early NAFLD symptoms become evident. The increase in MVs closely correlated with the histopathological findings. In addition, circulating MVs were enriched in miRNA-122 and miR-192-two microRNAs highly expressed in hepatocytes [49]. It has been shown that patients with NAFLD or NASH had high MV levels from natural killer (LK) lymphocytes and macrophages/monocytes; in those patients, MV correlated with severity of NASH (based on histology) [50]. Even though MPs derived from inflammatory cells are not specific for liver diseases, it has been shown that, using a proteomic approach, it is possible to tell apart MPs released because of free fatty acids oxidation-induced lipotoxicity, then originating from NASH [49].

Lastly, an interesting study has demonstrated that NASH could be diagnosed by the analysis of volatile organic compounds (VOCs) in the exhaled breath, using liquid chromatography and mass spectrometry. Verdam et al. studied VOCs from 65 obese patients undergoing bariatric surgery and liver biopsy and they found that three particular VOCs (*n*-tridecane, 3-methyl-butanonitrile, and 1-propanol) were typical of NASH and allowed to discriminate it from NAFLD. New analyses are needed to validate this method, but given its simplicity, it could be the election technique for NASH diagnosing in the near future [51].

## 5. Pathogenesis of NASH: Two-Hit versus Multiple-Hit Hypothesis

During the past years, different theories have been formulated about NAFLD onset and progression to NASH, from the traditional “two-hit” to the novel “multiple-hit” hypothesis. According to the two-hit hypothesis, the intrahepatic fat accumulation, triggered by sedentary lifestyle, bad nutritional habits and insulin resistance, represents the first hit [52]. The second hit consists in a lipid-induced over-production of reactive oxygen species; it worsens this scenario because of the interplay of various insults, such as: cytokine-mediated inflammation, free fatty acid oxidation, apoptosis, necroinflammation and fibrosis [53].

**Figure 1:**
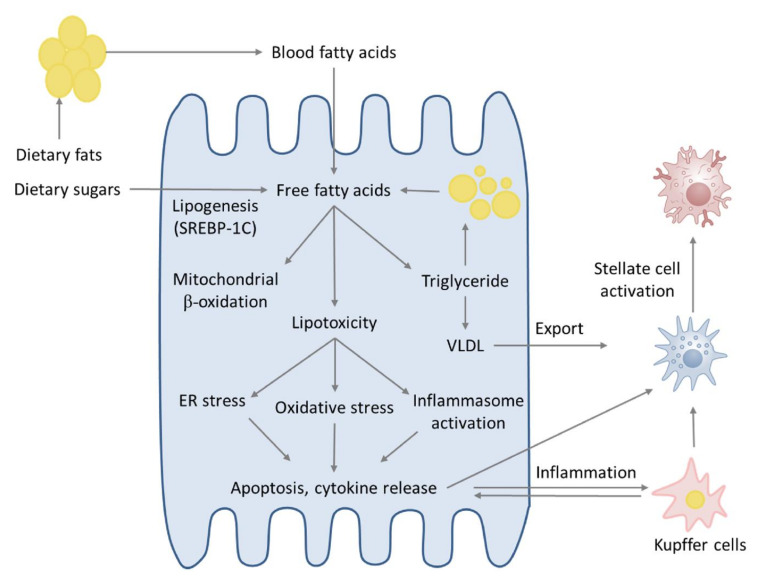
Non-alcoholic steatohepatitis (NASH) pathogenesis. Free fatty acids have a key role in the pathogenesis of NASH. Free fatty acids primarily originate from lipolysis of triglycerides in adipose tissue; insulin resistance may contribute to NASH through a lipolysis dysregulation. The other major contributor to the free fatty acid liver accumulation is de novo lipogenesis (DNL), resulting from hepatocyte-mediated conversion of fructose to fatty acids. Fatty acids, in hepatocytes, are directed toward mitochondrial beta-oxidation or re-esterification to form triglycerides. Triglycerides can be exported into the blood as VLDL or stored in lipid droplets. When the beta-oxidation or triglyceride conversion of fatty acids is overwhelmed, lipotoxicity occurs, leading to ER stress, oxidative stress and inflammasome activation. These processes are responsible for NASH development, resulting in hepatocellular injury, inflammation and fibrosis.

However, the two-hit hypothesis, by general consensus, is currently considered too simplistic to describe the complexity of human NASH development. In fact, it has been shown that oxidative stress does not necessarily follow lipid accumulation and it is able per se to induce steatosis [54,55]. Following this reasoning, it would be more correct to say that these events occur concurrently and they synergistically contribute to the development and progression of the disease as an integrated mechanism of different processes so-called “multiple-hit hypothesis” [2]. Hebbard & George explain the multiple-hit hypothesis as an “integrated response” of the organism to the combination of high-calorie diets, excessive food consumption and sedentary lifestyle in a genetically predisposed host. Altogether, these factors could lead to metabolic syndrome and obesity. After excessive food consumption, an imbalance of gut microbiota occurs and an increase in bacterial products is found in the portal circulation, activating the innate immune system. These events are accompanied by insulin resistance in the muscle, one of the key processes in the onset of the NAFLD-NASH, in response to the increased levels of circulating free fatty acids [56]. Insulin resistance causes the increase in hepatic de novo lipogenesis (DNL) and an imbalance in adipose tissue lipolysis, producing a great amount of circulating fatty acids that are conveyed to the liver. Moreover, insulin resistance causes the adipose tissue to release adipokines and inflammatory cytokines [57]. At the same time, hepatic fat accumulation leads to lipotoxicity, a condition promoting oxidative stress and affecting mitochondrial and endoplasmic reticulum physiological functions [58]. Altogether, these processes lead to hepatic chronic inflammation accompanied by cell death, hepatic stellate cell (HSC) activation and fibrosis (Figure 1). Thus, the assumption that steatosis always precedes inflammation is not completely correct, because the NASH can also be the initial hepatic injury: it is the timing and the combination of the various insults that determines whether steatosis or NASH will occur [59].

## 6. Molecular Pathways Involved in NAFLD

The main hallmark of NAFLD is the hepatic overload of fatty acids. Lipid accumulation can originate by different insults: it has been estimated that 2% of lipid accumulation is drug-induced, i.e., by some chemotherapeutic agents such as irinotecan and oxaliplatin [60]; another 2–5% result from endocrine dysfunctions, such as hypothyroidism, growth hormone deficiency and polycystic ovary syndrome [61]; HCV viral infection could also be involved [62]; however, the major cause of liver fat accumulation is caloric overload. Nevertheless, all these causes share an impairment of the molecular pathways involved in lipid metabolism, such as fatty acid uptake, DNL, fatty acid oxidation and fatty acid transport by VLDLs [63].

### 6.1. Fatty Acid Uptake

Until recently, fatty acid transport was believed to occur only by passive diffusion. In fact, lipids, being hydrophobic molecules, can bind to albumin to be transported into plasma and then released to diffuse through membranes [64]. However, this transport mechanism has emerged to be insufficient to clarify lipid distribution throughout body tissues, because fatty acid delivery would depend only on fatty acid gradient between the extracellular and intracellular environment, resulting in a difficult regulation [65]. Facilitated transport has been suggested as an alternative mechanism and several proteins have been recognized to be involved in this process, such as fatty acid transport proteins (FATPs), the fatty acid translocase/cluster of differentiation 36 (FAT/CD36) and fatty acid binding proteins (FABPs) [66]. It has been demonstrated that FATP2 and FATP5 are the two isoforms present in mice liver [67], and their knockdown or knockout is associated with a decreased fatty acid uptake [68]. FAT/CD36 is an integral membrane glycoprotein, expressed in a variety of cells, such as adipocytes, macrophages and hepatocytes [66]. Mice fed with a high-fat diet (HFD) develop steatosis and present an increase in CD36 mRNA and protein expression. On the contrary, liver-specific deletion of CD36 shows decreased hepatic lipid concentration in both genetic and diet-induced steatosis models [69]. FABPs are proteins located within membranes, as well as in cytosol and nucleus, so they play an important role in fatty acid uptake and intracellular trafficking. FABP1 is also called L-FABP because it is mainly expressed in liver. FABP1 suppression has been correlated to the reduction of steatosis, inflammation and oxidative stress, suggesting that FABP1 downregulation may slow down the progression of NAFLD into NASH [70].

DNL is responsible for the synthesis of new fatty acid chains in the liver, starting from acetyl-CoA [71]. Since several factors are involved in DNL, it is important to understand the mechanism behind this process. Initially, ACC adds a carboxyl group to acetyl-CoA producing malonyl-CoA, which in turn is converted into palmitate by fatty acid synthase (FAS). At this point, the new fatty acid molecule is subjected to consecutive transformations performed by desaturases and elongases, before finally being esterified and stored as triacylglycerol (TAG) or exported as VLDL [68]. However, an unjustified increase in DNL, coupled with an impaired oxidation rate, leads to steatosis and steatohepatitis [72]. Several studies have demonstrated that an elevated rate of hepatic DNL is a characteristic feature of NAFLD both in animal models and in patients [73,74].

When glucose levels are in short supply, mitochondria, as well as peroxisomes, oxidize lipids in order to provide energy in the form of reducing agents, such as nicotinamide adenine dinucleotide (NADH) and flavin adenine dinucleotide (FADH2) [75]. Fatty acid oxidation (FAO) is a cyclic process in which fatty acids are shortened, releasing acetyl-CoA units after each cycle [76]. Firstly, a fatty acid chain is converted into fatty acyl-carnitine and then transported into the mitochondria by the carnitine palmitoyltransferase (CPT)-1 and carnitine acylcarnitine translocase (CAT), respectively. Then, CPT2 converts fatty acyl-carnitines to fatty acyl-CoA-ester. One of the acyl-CoA-dehydrogenases converts the acyl-CoA-ester in a trans-2-enoyl-CoA, which in turn is hydroxylated into beta-hydroxyacyl-CoA and dehydrogenated into 3-keto-acyl-CoA, before the final cleavage that produces a shortened chain plus an acetyl-CoA. The acetyl-CoA produced can enter the tricarboxylic acid cycle and the reducing agents convey the electrons to the electron transport chain [67]. One of the main regulators of this process is the peroxisome proliferator-activated receptor (PPAR) α [77] as described below; however, other factors are also involved in fine-tuning the process. For example, the malonyl-CoA obtained in the first step of DNL inhibits CPT1, which is required to import fatty acid into the mitochondria for FAO [78]. Cytochromes are also able to oxidize fatty acids. It has been demonstrated that the cytochrome CYP4A14 is significantly upregulated in both patients and animal NAFLD models, causing lipid accumulation. On the contrary, the depletion of CYP4A14 gene attenuated the development of steatosis in mice fed with a high-fat diet or a methionine/choline-deficient diet [79]. As a result of lipid hyperaccumulation, an increased oxidation by peroxisomes and cytochromes occurs, resulting in higher levels of reactive oxygen species (ROS) and toxic molecules, finally leading to inflammation and disease progression, when the reducing agents cannot counterbalance oxidative stress [80].

Once fatty acids are synthetized through DNL, they are stored into water-soluble VLDLs before being secreted into plasma and transported to peripheral tissues, such as skeletal muscle, heart and adipose tissue [81]. VLDLs are assembled in the endoplasmic reticulum in a two-step process, both mediated by the microsomal triglyceride transfer protein (MTTP). In the first step, a newly synthesized apolipoprotein B-100 (apoB-100) is lipidated and a small, dense VLDL precursor is produced. During the second step, this particle is delivered to the Golgi apparatus where it is merged with a large triglyceride to generate a mature triglyceride-rich VLDL, ready to be secreted into plasma [82]. Even though the triglycerides contained into VLDL can vary, only one molecule of apoB-100 is needed for VLDL export. For these reasons, MTTP and apoB-100 are considered the key components of this process. In fact, it has been observed that, in patients with genetic defects in the apoB or MTTP gene, the triglycerides export is impaired, causing steatosis [83]. Moreover, NAFLD patients are characterised by an increased secretion of slightly larger VLDLs when compared with healthy subjects, suggesting that more triglycerides are incorporated in these VLDL particles [84]. However, research on animal models has clarified that very large VLDLs cannot be secreted because they are too large to move throughout the sinusoidal endothelial pores, thus exacerbating lipid retention [85].

### 6.2. mTOR Signalling Pathway

The mammalian target of rapamycin (mTOR) is a 289-kDa serine/threonine kinase, belonging to the phosphoinositide 3-kinase (PI3K)-related family. In association with other components, it constitutes two different multi-protein complexes, mTOR complex 1 (mTORC1) and mTOR complex 2 (mTORC2), which control cell metabolism, growth, proliferation and survival [86].

After food intake, insulin binds to the insulin receptor. The activated receptor enrols the insulin receptor substrate (IRS), activating PI3K. PI3K, in turn, phosphorylates phosphatidylinositol 4,5-bisphosphate (PIP2), converting it into phosphatidylinositol (3,4,5)-trisphosphate (PIP3), which stimulates phosphoinositide-dependent protein kinase 1 (PDK1), resulting in the activation of Akt/protein kinase B (PKB). PKB, by inhibiting the mTORC1 negative regulator tuberous sclerosis complex (TSC), a heterodimer composed by TSC1 and TSC2, finally activates mTORC1 [86,87]. mTORC1 coordinates DNL and glucose metabolism by promoting the activation of SREBP1c and the hypoxia-inducible factor-1 α (HIF-1 α) [88,89]. SREPB1c is a basic helix-loop-helix transcription factor synthetized as an inactive precursor anchored to the membranes of the endothelial reticulum (ER), where it interacts with a sterol sensor, the SREBP cleavage-activating protein (Scap). In case of lipid shortage, SREBP1c/Scap complex is delivered to the Golgi apparatus and, after a two-step cleavage, the mature form of SREBP1c migrates into the nucleus [90]. Here, SREBP1c, by binding to sterol regulatory element (SRE) sequences in the promoter of several genes, induces the expression of genes required for maintaining the homeostasis of cholesterol and lipids, such as ACC1 and ACC2, FAS and steroyl-CoA desaturase (SCD) [91]. SREBP1c regulation is managed by mTORC1 through several modalities [87]; in HepG2 cells treated with palmitate to induce intracellular lipid accumulation, mTORC1 directly stimulates SREBP1c via its downstream effector S6 kinase 1 (S6K1) [92]. However, different S6K1-independent mechanisms were reported for SREBP1c activation [93]. In fact, mTORC1 induction causes ER stress, leading to the consequent activation of hepatic SREBP1c [94]. mTORC1, by phosphorylating the CREB-regulated transcription coactivator 2 (CRTC2), increases SREBP1c trafficking from the ER to the Golgi apparatus [95]. In addition, mTORC1 promotes SREBP-1 nuclear import also through Lipin-1 phosphorylation. Lipin-1 is a phosphatidate phosphatase able to produce triglycerides and phospholipids through a diacylglycerol-mediated mechanism. Lipin-1 has multiple phosphorylation sites, including the mTORC1 rapamycin-sensitive sites. The phosphorylated Lipin-1 resides in the cytosol, however, when it is no longer phosphorylated, Lipin-1 moves into the nucleus and, acting as a transcriptional co-regulator, down-regulates SREBP1c protein and consequently its target genes [96].

### 6.3. PPAR-α

PPAR-α, together with PPAR-γ and PPAR-β/δ, is a ligand-activated transcription factor belonging to the NR1C nuclear receptor subfamily [97]. To exert its function, PPAR- α heterodimerizes with the retinoic X receptor (RXR) and binds to the PPAR response elements (PPREs) present in the promoter region of various genes. The core sequence consists of two direct repeats of AGG(A/T)CA, separated by one nucleotide (DR-1) [98]. PPAR-α is ubiquitously distributed, especially in the liver, heart, brown tissue and skeletal muscle, which are characterized by high fatty acid oxidation rates [99]. In fact, PPAR-α, acting as a nutritional sensor for fatty acids, is mainly known to induce the hepatic expression of genes implicated in fatty acid import, including carnitine palmitoyltransferases and the solute carrier protein SLC22A5 [100,101]. Direct targets of PPAR-α are also genes involved in mitochondrial and peroxisomal β-oxidation, for example acyl-CoA dehydrogenase very long chain (ACADVL) and acyl-CoA oxidase 1 (ACOX-1), respectively [102].

The activity of PPAR-α in lipid regulation is multifaceted, because it also balances the uptake of fatty acids, as well as the DNL, ketonegenesis and triglyceride export in both feeding and fasting conditions [103,104]. During fasting conditions, PPAR-α induces mitochondrial delivery of fatty acids deriving from adipose tissue lipolysis through transcriptional up-regulation of fatty acid transport proteins such as FATP1 and FAT/CD36 as well as the intracellular transporter L-Fabp, whose gene contains a PPRE sequence in the promoter region [105]. Moreover, PPAR-α has been found to control lipoprotein metabolism, because of the presence of a PPRE sequence within the lipoprotein lipase (LPL) promoter [106]. PPAR-α attenuates VLDL assembly, which results in the reduction of plasma triglycerides [107]. In addition, the administration of PPAR-α agonists was related to the decrease of the LPL inhibitor apolipoprotein C III in patients affected by metabolic syndrome, type 2 diabetes and dyslipidemia [108]. In severe fasting, PPAR-α regulates ketogenesis, up-regulating the rate-limiting enzyme hydroxymethylglutaryl-CoA synthase (HMGS), triggering acetyl-CoA conversion into ketone bodies and providing energy for extrahepatic tissues [109]. Furthermore, PPAR-α exhibits a strong anti-inflammatory activity by suppressing NF-𝜅B [110]. PPAR-α directly inhibits pro-inflammatory signalling pathways via protein–protein interactions; in human primary hepatocytes, ligand-activated PPAR-α attenuated interleukin 1(IL-1), IL-6 and TNF-α in vitro and in vivo [111].

### 6.4. The Interplay between mTOR and PPAR-α

Lipid pathways should not be considered as separate processes but as intertwined mechanisms; in fact, although each pathway has specific self-regulation mechanisms, some factors mutually coordinate each other. As mentioned before, PPAR-α works both in fed and fasting states [105]. In the fed condition, PPAR-α controls DNL at several levels. Human SREBP1c contains a DR-1 element on its promoter, thus PPAR-α directly interacts with it [112]. In mice, even if PPAR-α agonists positively regulate SREBP1c target genes such as FAS and ACC without being PPAR-α targets, it has been observed that a PPRE is present in the promoter of *Scd-1* and on delta 6 desaturase [113,114]. Moreover, the administration of PPAR-α agonists is related to the proteolytic cleavage of SREBP1c precursor from the membrane, without altering SREBP1c mRNA levels [113]. PPAR-α controls SREBP1c transcription also indirectly, via cross-regulation with LXR-signalling pathway, because LXR, a direct target of SREBP1c, contains on its promoter a PPRE sequence [115].

During starvation, PPAR-α stimulates FAO and ketogenesis. Thus, the transition of PPAR-α activity according to the fed/fasting condition can be regulated by different kinases. In the fed state, PPAR-α is transactivated by insulin-mediated MAPK and glucose-mediated PKC; while in fasting, PPAR-α activity is increased by PKA, promoting FAO and producing glucose and ketone bodies [116]. Another mechanism to modulate PPAR-α is mediated by mTORC1. In the fed state, mTORC1 is activated by the insulin-PI3K pathway [87]. This inhibits PPAR-α target genes expression, because the co-repressor of PPAR-α, NCoR1, is located in the nucleus. On the contrary, during fasting, mTORC1 is inhibited, NCoR1 is exported into the cytoplasm, and PPAR-α is activated. Furthermore, mTORC1 controls ketone body production in response to starvation: the loss of TSC1 complex, which inhibits mTORC1, causes a fasting-resistant expansion in liver size and a marked defect in ketogenesis because of a decrease in ketogenic gene expression. The loss of Raptor, one mTORC1 component, has opposite effects [117].

The activation of mTORC1 can occur by stimuli other than insulin, such as amino acids and simple sugars, or by the activation of different kinds of receptors, like cytokine receptors, AMPA and NMDA receptors. In neurons also several G-protein coupled receptors (GPCRs) activate mTORC1, including the metabotropic glutamate receptor subtype 5 (mGluR5), dopaminergic D1 and D3 receptors and GABA_B_ receptors [93,118].

Most of the knowledge about mGluR5 emerges from studies performed in the central nervous system (CNS), where mGluR5 is mainly localized [119]. This receptor is coupled to G_αq_/G_11_ and activates phospholipase C β (PLC β), leading to the hydrolysis of phosphoinositides and the formation of inositol 1,4,5-trisphosphate (IP3) and diacylglycerol [120]. mGluR5 is also able to stimulate PI3K, activating its downstream cascade including Akt/PKB, PDK1 and mTOR [121]. It has been reported that in mouse hippocampal slices the pharmacological activation of mGluR5 triggers the PI3K-mTOR pathway [122]. In their work on FXS mouse model, Ronesi and Huber clarified that PI3K-mTOR pathway was stimulated through the mGluR5 scaffolding protein Homer [123]. Furthermore, the blockade of mGluR5 in a mouse model of Huntington disease prevents the progression of this disorder, by modulating mTOR cascade [124].

The mGluR5 activation has been correlated with the modulation of central reward pathways of food intake and energy balance in rodents [125]. In fact, in mGluR5 knockout mice, as well as in mice administered with MTEP, an mGluR5 antagonist, a decrease in weight and food intake was observed. In addition, also in diet-induced obese rats, MTEP treatment reduced food ingestion and avoided weight gain [125].

mGluR5 was found also in HSCs, where it is involved in promoting alcoholic steatosis [126]. In fact, glutamate is produced in excess within hepatocytes with elevated oxidative stress and exchanged with cysteine by the antiporter xCT to reinforce antioxidant defence. Extracellular glutamate hyperactivates mGluR5 onto HSCs, stimulating the production of 2-arachidonoylglycerol (2-AG), which in turn drives DNL in hepatocytes. Nevertheless, the genetic or pharmacological blockade of the antiporter or the mGluR5 mitigates steatosis [126].

The monosodium L-glutamate (MSG) is one of the most employed flavor enhancers (food additive number E621) all over the world [127]. MSG has been shown to cause obesity and dyslipidemia, inflammation and hepatic steatosis, through the increase in fatty acid oxidation genes [128]. MSG administration for 6 and 12 months in mice leads to NAFLD and NASH-like histology, respectively [129]. Very recently, it has been demonstrated that, in an in vitro model of steatosis, mGluR5 has a role in lipid homeostasis. HepG2 cells treated with a mixture of oleic and palmitic acid (molar ratio 2:1) presented an increase in fatty acid overload, positively correlated with increased SREBP1c and negatively correlated to PPAR-α. The blockade of mGluR5 restores these parameters to control conditions. Considering the existing interplay between mGluR5 and mTOR in other districts, it has been suggested that mGluR5 may activate mTOR also in the liver, resulting in the promotion of lipid accumulation [130].

## 7. Management of NAFLD and NASH

To date, there are no specific pharmacological therapies recommended for the treatment of NASH [37]. The treatment for patients suffering from NAFLD and NASH consists of recommendations aimed at changing lifestyle and nutrition, in particular weight loss and physical exercise. Indeed, patients who obtained a weight loss of about 10% displayed a reduction in liver fibrosis and NASH severity [131]. Unfortunately, the majority of patients fail to maintain weight loss; then, these recommendations are considered to be of little effectiveness [132].

Different pharmacological approaches have been showing some efficacy in the management of NAFLD and NASH (Table 1). For example, since lipid accumulation is promoted by insulin resistance, some pharmacological medications used in type II diabetes, such as biguanides and thiazolidinediones (TZDs), have been employed. The biguanide metformin improves insulin sensitivity through activation of AMP-activated kinase and blocking gluconeogenesis [133], but it does not induce an histological amelioration, especially in NASH [134]. In fact, since metformin is not able to restore adiponectin concentration in a short time, its effect on liver fat is weak [37]. A comparative analysis conducted by Zhou and colleagues showed that the treatment with metformin reduced the risk of HCC; on the contrary, insulin administration was associated with an increased risk for HCC [135]. However, the decrease in the risk of HCC is very limited and still relative to retrospective studies; therefore, current data are not sufficient for an evidence-based recommendation [37].

Conversely, -PPAR-γ agonists, such as pioglitazone and rosiglitazone, are considered a promising treatment for NASH. Pioglitazone has been studied in several randomized clinical trials and it seems to reduce significantly steatosis, inflammation, hepatocellular ballooning and fibrosis [136,137]. Rosiglitazone did not induce histological improvements, probably because, differently from pioglitazone, it does not act by additional mechanisms, i.e., by interacting with the mitochondrial target of thiazolidinediones (mTOT) [138]. Despite the promising results obtained with pioglitazone, its use is limited in patients with NASH because of TZD-induced adverse effects, including weight gain, fluid retention, osteopenia and increase in fracture risk in elderly women [37,138]. Rosiglitazone was also associated with increased risks of vascular disease, precipitation or exacerbation of congestive heart failure [139].

Recently, a new therapeutic approach consisting in the use of glucagon-like peptide-1 (GLP)-1 analoguesliraglutide has been proposed. The intestinal-derived incretin hormone GLP-1 is produced after proteolytic cleavage of proglucagon and it acts by stimulating insulin and blocking glucagon secretion and suppressing appetite [140]. Unlike the endogenous GLP-1, liraglutide shows a longer elimination half-time [141], which promotes weight loss [142]. This medicament is approved for the treatment of type II diabetes, but a phase II trial conducted in four UK medical centers showed that liraglutide induces histological improvements in NASH with a daily injection [37,143]. In another placebo-controlled, phase II trial, NASH resolution with no worsening of fibrosis was reported in 59% of patients receiving 0.4 mg of the GLP-1 analogue semaglutide versus 17% in the placebo group (*p* < 0.001); however, no significant improvement of fibrosis was observed between groups [144].

Therapeutic medicaments targeting oxidative stress and inflammation, such as vitamin E, have also been employed in NASH patients. Vitamin E has been shown to be effective especially in pediatric patients with mild NAFLD/NASH; currently, available studies do not recommend vitamin E for NASH treatment in adults as it is marginally better than placebo and significantly less effective than glitazones [145]; its non-specific interaction with cardiac [146] and oncologic disease [147] should also be taken into consideration.

Further pharmacological medicaments for the treatment of NASH are hydrophilic bile acids (BAs), which can modulate both glucose and lipid handling [148] through the activation of farnesoid X receptor (FXR) [149,150] and Takeda G protein-coupled receptor 5 (TGR5) [151], displaying a significant anti-inflammatory activity [152]. Several agonists for both receptors have been synthesized; for example, the ursodeoxycholic acid (UDCA), investigated in various randomized controlled trials, displayed only biochemical, but not histological benefits [37]; differently, the FXR agonist obeticholic acid (OCA) is now the prototype for this kind of compounds because of its positive effects on insulin resistance [153]. OCA administered at 25 mg per day doses, as during the phase II of the experimental trial, induced pruritus, an increase in the level of low-density lipoprotein (LDL) and cholesterol, in some individuals [37,154,155]; then, phase III of the clinical trial was focused on obtaining a more tolerable dose of the drug in the long period, without losing effectiveness.

## 8. Drugs Currently under Development for NASH Therapy

New therapeutic strategies targeting receptors involved in fatty acid metabolism are currently under preclinical or clinical development (Table 2). Some anti-NASH drugs target metabolic pathways involved in hepatic fat accumulation; these drugs include: FXR agonists, PPAR agonists, de novo fat synthesis inhibitors such as the pan acetylcarboxylase (ACC) inhibitor MK-4074 and analogues of fibroblast growth factor (FGF). Some of these drugs are currently under development in phase III clinical trials [156].

Small-molecule FXR agonists, when compared with bile acids, have a somewhat reduced ability to induce side effects, such as increasing cholesterol and pruritus, because of a different molecular structure [138]. OCA was approved by FDA for the treatment of primary biliary cholangitis (PBC) in 2016; recently, studies have been conducted to demonstrate OCA efficacy in reducing future risk of NASH complications, including liver failure, cirrhosis and hepatocellular carcinoma. In the FLINT phase IIb trial, improvements in the histologic features of NASH and in aminotransferases were observed [155]; however, doubts remained about its long-term efficacy and safety; in particular, OCA was associated with an increase in insulin resistance [157]. The randomized global phase III trial REGENERATE (registration no. NCT02548351), initiated by Intercept Pharmaceuticals (San Diego, CA, USA), was aimed to assess the impact of OCA treatment on NASH and fibrosis. This trial successfully demonstrated an improvement of fibrosis in 18–23% of patients but failed in resolving NASH [158], resulting in FDA rejection [159]. Another phase III trial evaluating the efficacy and safety of OCA in patients with compensated cirrhosis due to NASH was initiated in 2017 (REVERSE; registration no. NCT03439254). OCA is considered the drug currently at the most advanced development stage and the one likely to be the first anti-NASH drug on the market [156].

PPAR-α agonists are known to have positive effects in controlling fatty acid oxidation and inhibiting NFkB-induced inflammatory factors [105]; PPAR- agonists, instead, intensify lipid transport and oxidation, increase insulin sensitivity and decrease gluconeogenesis [160]; they have also shown anti-inflammatory properties [161]. Elafibranor, combining PPAR- and PPAR- agonism, has shown interesting properties in different rodent models of NAFLD and NASH, decreasing liver fatty acid accumulation and also attenuating pro-inflammatory and pro-fibrotic factors activity [162]. In a randomized, double-blind placebo-controlled phase II trial on NASH patients, Ratziu and colleagues demonstrated that the administration of 120 mg/day of elafribranor for one year ameliorated the metabolic traits of NASH; the treatment was well tolerated and was not associated with higher cardiovascular risk [163]; however, elevation of serum creatinine occurs, potentially limiting its use in patients with renal insufficiency [164]. Currently, elafribranor is under evaluation in a phase III trial (RESOLVE IT; NCT02704403) [138].

Acetyl-CoA carboxylases (ACC1 and ACC2) inhibitors are key enzymes in the fatty acid elongation process. In a small Phase II study, the pan-ACC inhibitor MK-4074, when administered to steatotic subjects for 1 month, reduced DNL but induced hypertriglyceridemia. The same authors demonstrated, in fact, that a decrease of polyunsaturated fatty acids occurs in mice lacking ACC enzymes in the liver, inducing, as a feedback effect, an increase in sterol regulatory element-binding protein (SREBP)-1c activity with the consequent increase in serum triglycerides [165].

To counteract apoptosis, which promotes inflammation and fibrosis, some caspase inhibitors are currently being tested. One of them is emricasan, a pan-caspase inhibitor that was active against caspase-3 and caspase-8 in mice fed with high-fat diet. The inhibition of the apoptotic cascade also induced a reduction in liver injury and inflammation [166]. In a Phase III clinical trial, despite a reduction in activated caspases indicative of pharmacodynamic activity, emricasan did not improve portal pressure or clinical outcomes in patients with NASH-derived cirrhosis [167].

The inhibition of regulatory pathways involved in inflammatory response has been taken into account, such as MAP-kinases and NFkB pathways. Unfortunately, these factors play important roles in many different vital processes, so their inhibition could result in many side effects. At the moment, the Phase II clinical trial for selonsertib, inhibitor of the apoptosis signal-regulating kinase-1 (ASK-1) in the MAP-kinases cascade, did not reveal dangerous side effects and induced an amelioration of NASH and fibrosis as well as of serum biomarkers of apoptosis and necrosis in patients [168]. In a Phase III clinical trial, selonsertib led to a dose-dependent reduction in hepatic phospho-p38 expression, indicating target engagement; however, it did not reduce fibrosis in patients with NASH and advanced liver scarring after a 48-week treatment [169].

## 9. Experimental Models for the Study of NAFLD and NASH

### 9.1. In Vitro Models

In vitro models (Table 3) are considered important tools for the investigation of the molecular mechanisms involved in the pathogenesis of many hepatic conditions; however, a simplistic set up, such as a monocell culture, is a limit when investigating NAFLD/NASH progression. In fact, many hepatic cellular subtypes, including parenchymal and non-parenchymal cells, contribute to NASH pathogenesis and development, such as: HSCs, Kupffer cells, sinusoidal endothelial cells and cholangiocytes [170,171,172]; consequently, a monocell culture is not suitable for the investigation of complex interactions occurring between different cell types [138]. Nonetheless, monocell cultures using human or murine primary cell cultures or hepatoma cell lines, are generally considered suitable instruments for the investigation of a specific intracellular pathway. Human primary hepatocytes are ideally preferred to immortalized and hepatoma cells such as HuH7 and HepG2 cell lines, since hepatoma cells are considered poor models of liver metabolic function [173]. However, human primary cells have a limited lifespan, an unstable phenotype and are not easily available for practical and ethical reasons [174]. To reproduce fat accumulation in hepatocytes, cells are incubated with oleate [175,176], palmitate [177] or both [130,178]. Furthermore, in vitro cultures of sinusoidal endothelial cells (SECs) have been used for the investigation of the molecular changes occurring under abnormal shear stress; in particular, a microfluidic setup is preferred as it recreates the shear stress of fluid flow inducing nitric oxide (NO) synthesis in SECs [179].

Simultaneous co-cultures were developed in the attempt to reproduce the interplay between hepatocytes and HSCs; the two cell types are seeded together allowing the investigation of cell-to-cell interactions. This approach reproduces more accurately the biochemical response to fat accumulation [180,181]. Transwell culture represent a similar approach in which two or three cell types are co-cultured into different surfaces sharing only the culture media [182].

To overcome the limitations of 2-D co-culture models, new in vitro models were proposed. In particular, the 3-D in vitro models have generated great attention since they represent more closely the in vivo situation with respect to morphology, adhesion, cell–cell interactions. Typically, for 3-D sferoids, human hepatoma or murine primary hepatocytes are used [183,184]; differently, 3-D organoids are cellular clusters derived from induced pluripotent stem cells (iPSCs), embryonic stem cells (ESCs) or tissue-resident progenitor cells, capable of self-renewal and self-organization [185]. Recently, iPSCs were shown to differentiate into hepatocyte organoids, recapitulating some aspects of NAFLD and NASH in vitro when treated with fatty acids, such as lipid accumulation and fibrosis [186]. In the future, patient-derived liver organoids may help in devising patient-tailored therapies [185].

### 9.2. In Vivo Genetic Models

**Figure 2:**
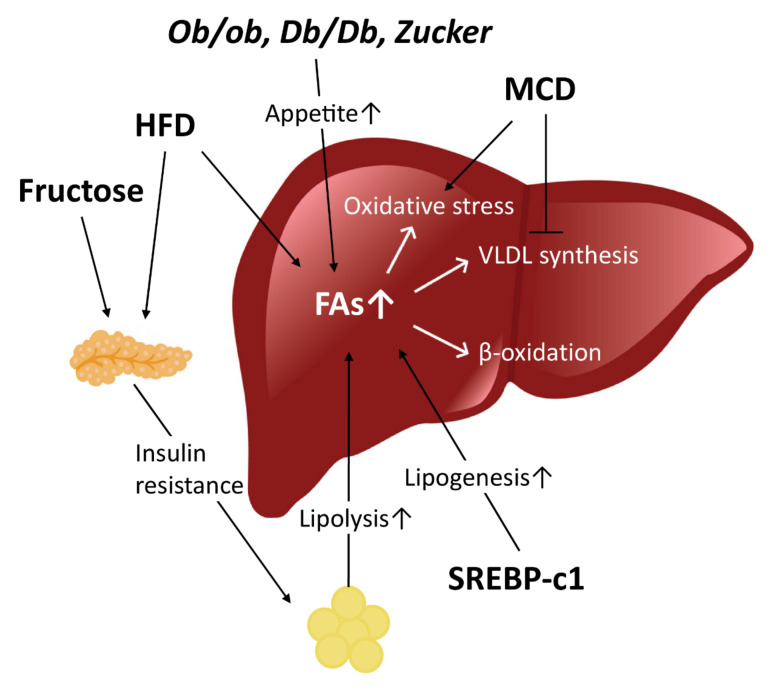
Mechanism of NAFLD pathogenesis in in vivo models. FA accumulation has a key role in NAFLD and NASH development. In *ob/ob*, *Db/Db* and Zucker models, a leptin-related increase in appetite results in insulin resistance and FA accumulation. HFD and fructose-rich diets induce insulin resistance and, secondarily, liposysis; HFD also causes a higher FA intake. In SREBP-c1 genetic model, FA increase is caused by a higher level of lipogenesis. MCD diet inhibits VLDL synthesis and export, as well as a GSH deficiency, leading to oxidative stress. ↑ indicates an increase. HFD, high-fat diet; SREBP-1c, sterol regulatory element-binding protein 1c; MCD, methionine and choline deficient; VLDL, very low-density lipoprotein.

Many different in vivo models have been developed for NAFLD-NASH in the attempt of mimicking the characteristics of human pathology as much as possible, and, among them, two different types of animal models are prevailing: the genetic and the nutritional models (Figure 2, Table 4) [47].

Since steatosis is the result of lipid intrahepatic accumulation, genetic models for NAFLD and NASH are obtained by engineering the genes strictly involved in this process [56]. The main defect of these models is the lack of inflammation and fibrosis. However, this condition is obtained by a second insult, such as the administration of a modified diet [187]. The most common genetic models to study NAFLD and NASH are listed below.

*Ob/ob* mice. *Ob/ob* mice exhibit a spontaneous mutation in the leptin gene that makes them unable to produce leptin in white adipose tissue. Normally, after secretion, leptin regulates the feeding behavior and energy bursts, promoting reduced food intake and increasing energy metabolism. In the *ob/ob* mice, the lack of interaction between leptin and its receptor makes these mice hyperphagic, extremely obese and inactive [188]. In addition, in animals older than 3–4 weeks, an altered metabolic profile is observed, leading to hepatocyte lipotoxicity and lipoapoptosis. *Ob/ob* mice display steatosis but they do not progress to NASH, so they are suitable for studies on the interaction between insulin and leptin, type-2 diabetes and NAFLD [138,189].

*Db/db* mice. These mice present a spontaneous mutation in the leptin receptor gene (*Ob-Rb*), so, even though they have normal leptin levels, they are resistant to its effects, showing the same features of *ob/ob* mice; the concomitant administration of an MCD diet for 4 weeks promotes severe inflammation and fibrosis [190]. Differently from *ob/ob* mice, *db/db* mice develop NASH when fed with a hypercaloric diet [191,192].

Zucker rats. One of the most common genetic model in rats, Zucker rats (*fa/fa*) are carriers of a spontaneous mutation in the leptin receptor (*fa* allele), leading to a reduced affinity for leptin. They show the same metabolic picture of *db/db* mice developing severe obesity and hyperleptinemia, hyperphagia, inactivity and insulin resistance; they display extensively diffused macro and micro-vesicular steatosis, mainly in the periportal area [193]. As in *db/db* mice, NAFLD in Zucker rats do not spontaneously progresses into steatohepatitis and a “second hit” is required, such as a high-fat diet or the combination of an MCD diet with a HFD [188,194].

SREBP-1c transgenic mice. In mammals, SREBPs play a pivotal role in controlling intracellular cholesterol and fatty acids levels. SREBP-1c overexpression in transgenic mice results in severe insulin resistance and spontaneous steatohepatitis with oxidative stress at 30 weeks of age. When compared with wild-type mice, an increase in fatty droplets infiltration is observed, but not an increase in body weight [195].

### 9.3. In Vivo Nutritional Models

Nutritional models of NAFLD (Figure 2) are divided in two main groups: models with reduced lipid export (choline deficient, methionine-choline deficient) and models with an increased lipid import or synthesis in the liver (high-fat diets, high-fructose/-sucrose diets, combined diets). NAFLD patients rarely exhibit evident genetic defects; so, the use of dietary models of NAFLD is more clinically relevant to human disease than genetic models. Currently, the most common rat model of NAFLD uses high-fat diets [188].

Methionine and choline deficient diet (MCD). MCD diet is high in sucrose and fats and lacks methionine and choline, which are essential for lipid oxidation and very low-density lipoprotein (VLDL) production in the liver [196]. Methionine restriction also induces a reduction in intrahepatic GSH, leading to an increase in oxidative stress [197]. MCD diet-fed animals display accumulation of intrahepatic lipid and decreased VLDL synthesis, accompanied by body weight loss, decreased liver size and hepatic steatosis that, within a period of 8 weeks, progresses to NASH due to the high levels of oxidative stress and inflammation [198]. Activation of macrophages that infiltrate liver tissue, as well as an increase in nuclear factor κB (NFκB) and cytokine production also occur [199,200]. MCD animals are suitable for studies on lipotoxicity, inflammation, cirrhosis and hepatocellular carcinoma (HCC) [201].

High-fat diet (HF). This kind of nutritional model mimics the western-style diet. Animals are fed with a diet in which caloric intake (45–75%) originates mainly from fat, fructose and cholesterol, considered the main responsible in the induction of obesity, insulin resistance and hepatic injury. The degree of hepatic damage is not as severe as that induced by the MCD diet, but it resembles NASH as it is observed in humans, including inflammation and fibrosis development [56].

High-fructose diet. To mimic the effects induced by an excessive consumption of soft drinks and corn syrup, highly consumed in western countries and responsible of obesity development and NAFLD, a high-fructose diet was also formulated. Fructose intake has many destructive effects, such as the induction of DNL, oxidative stress and insulin resistance [202]. Moreover, it promotes intestinal bacterial overgrowth, which leads to increased gut-derived endotoxin levels in the portal circulation, activating Kupffer cells and producing inflammation. In fact, in mice fed with water supplemented with fructose (30%) an increase in hepatic triglycerides, simple steatosis and body weight are observed [203]. Supplementation of 20% fructose in male Wistar rats results in obesity, hypertension and hyperglycemia; increased levels of serum triglycerides, accompanied by lipid deposition without ballooning and inflammation, are also observed after 8 weeks [204]. C57BL/6 mice fed with a combined high-fat diet and high-fructose water, display, after 16 weeks, insulin resistance, obesity, NASH, fibrosis and increased hepatic oxidative stress, with respect to mice fed with high-fat diet only [205]. We can conclude, even though it is not possible to obtain the hepatic manifestation of NASH using a high-fructose diet alone, that NASH-like symptoms are obtained by combining a high-fructose diet with a HFD [187].

Cholesterol and cholate diet. Since cholesterol accumulation is associated with insulin resistance, worsening of cardiovascular risk and NAFLD to NASH progression, researchers tried to mimic this situation using diets enriched in cholate and cholesterol [56]. The cholate and cholesterol diet (1.25% and 0.5%, respectively) produces steatosis, inflammation, HSC activation, fibrosis and altered levels of ALT, which increase progressively from 6 to 24 week [206]; however, animals fed with this diet are not insulin-resistant; they actually tend to lose weight and have lower triglyceride levels, with respect to standard chow-fed mice [187].

## 10. Conclusions

Recently, an increasing trend has been observed for NAFLD, currently affecting 24% of the world’s population. A similar increasing trend has been found for NASH, whose prevalence has been estimated from a few biopsy series in a range between 1.5% and 6.45% [207]. According to the multiple-hit hypothesis, multiple events concur to the development of NASH, which is an integrated response of the organism to the combination of high-calorie diet, excessive food consumption and sedentary lifestyle in a genetically predisposed host. To date, there are no specific drugs recommended for the treatment of NASH; the most used medications include drugs used in type-2 diabetes, such as glitazones and GLP-1 analogues, but also antioxidants and bile acids. New drug candidates are currently under development, including ACC inhibitors, PPAR inhibitors, caspase inhibitors and FXR agonists. Many experimental models are currently being used for drug discovery and development. Multicellular spheroids and organoids are preferred to monocell culture for the study of NASH, because multicellular 3-D models represent more closely the interplay between the various hepatic cell types, as well as liver morphology and cell–cell interactions. Nutritional models, often administered to genetically modified animals, are the most used in vivo NASH experimental models. Obtaining more in-depth knowledge of the molecular pathways involved in NAFLD pathogenesis and in the progression from asymptomatic, simple steatosis to NASH, is of pivotal importance for the development of new drugs that may improve patient outcomes, as well as reducing the overall mortality and the demand for liver transplantation.

## Figures and Tables

**Table 1:** Current management of nonalcoholic fatty liver disease (NAFLD) and NASH.

Drug	Class	Pros	Cons
Metformin	Biguanide	Weight loss, improvement of insulin sensitivity	No histological improvement, not recommended in NASH patients
Pioglitazone	Thiazolidinediones	Reduction of steatosis, inflammation, hepatocellular ballooning and fibrosis	Weight gain, fluid retention, increased fracture risk in elderly women
Rosiglitazone	Thiazolidinediones	Reduction of steatosis, inflammation, hepatocellular ballooning and fibrosis	Increased risk for myocardial infarction, weight gain
Liraglutide	GLP-1 analogues	Weight loss, histological improvement of NASH	Not orally available, increased risk for pancreatitis
Vitamin E	Antioxidant	Improvement of steatosis, ballooning and inflammation	Limited effects in adults, only effective in pediatric patients with mild NAFLD
UDCA, OCA	FXR agonists	Amelioration of insulin resistance (OCA) and biochemical markers (UDCA)	Pruritus, increased levels of LDL and cholesterol

**Table 2:** Drugs currently at various stages of development for the therapy of NASH.

Drug	Class	Pros	Cons
Obeticholic acid	FXR agonist (different structure from bile acids)	Anti-inflammatory activity, improvement of insulin resistance	High cholesterol and pruritus
MK-4074	Pan-acetyl-CoA carboxylase inhibitor	Reduce DNL	Increase in SREBP-1c activity, resulting in high serum TG levels
Elafibranor	PPAR-α and PPAR-δ agonist	Inhibition NFkB-induced inflammation, increased lipid transport and oxidation, increased insulin sensitivity	Elevation of serum creatinine levels
Emricasan	Pan-caspase inhibitor	Antiapoptotic effects, consequent reduction of liver injury and inflammation	Portal pressure is not reduced in patients with cirrhosis
Selonsertib	Inhibitor of the apoptosis signal-regulating kinase-1 (ASK-1), MAPK inhibitor	Amelioration of serum biomarkers	No improvement of fibrosis

**Table 3:** In vitro experimental models for the study of NAFLD and NASH.

Models	Pros	Cons
Primary cell monocultures	Ideal model of liver metabolic function; simple and standardized setup	Limited availability; short-term culture; freshly prepared each time; not suitable for studies on fibrosis
Hepatoma cell monocultures	Easily available, long-term cultivation; simple and standardized setup	Altered expression of several enzymes and nuclear factors; not suitable for studies on fibrosis
2-D co-cultures	Improved model of NAFLD-NASH progression and interplay between hepatocytes and HSCs	Limited spatial interaction and morphology; lack of standardized conditions
3-D organoids and spheroids	Improved morphology and spatial interaction; reproduction of the interplay between two or three different cell types; auto organization and regeneration (spheroids)	Complex setup; not readily available; lack of standardized conditions

**Table 4:** In vivo experimental models for the study of NAFLD and NASH.

Model	Features	Pros	Cons
Genetic models			
*Ob/ob*	Leptin-deficient mice; obesity, insulin resistance and steatosis	Physiological model of simple NAFLD	NASH does not develop without a second insult
*Db/Db*	Mice with leptin receptor mutation; obesity, insulin resistance and steatosis	Physiological model of NAFLD	NASH does not develop without a second insult
Obese *fa/fa* Zucker	Rats with nonfunctional leptin receptor	Physiological model of NAFLD	NASH does not develop without a second insult
SREBP-1c	Mice with hepatocyte-specific SREBP-1c overexpression; insulin resistance, steatosis and fibrosis	Mild NASH phenotype	No weight increase
Nutritional models			
Methionine and choline deficient diet (MCD)	Steatosis, fibrosis, high oxidative stress and inflammation, body weight loss, decreased liver size	Fast induction time and progression to NASH.	Not suitable for investigating metabolic syndrome and insulin resistance
High-fat diet (HF)	Obesity, steatosis, steatohepatitis, insulin resistance	Physiological model of NAFLD/NASH with increased oxidative stress, collagen type I and α1(I) pro-collagen mRNA upregulation, increased levels of TNF-a and damaged mitochondria	Slower induction time
High-fructose diet	Obesity, steatosis, fibrosis, insulin resistance	Physiological model of NAFLD	Not possible to obtain the hepatic manifestation of NASH without adding an additional insult
Cholesterol and cholate diet	Steatosis, fibrosis	Histological features of NASH	No systemic insulin resistance, may induce body weight loss

## Abbreviations

2-AG	2 arachidonyl glycerol
ACADVL	acyl-CoA dehydrogenase very long chain
ACC	acetyl CoA carboxylase
ACOX-1	acyl-CoA oxidase 1
ADMA	asymmetric dimethylarginine
AF	autofluorescence
apoB100	apolipoprotein B 100
ASK-1	apoptosis signal-regulating kinase
BA	bile acid
CH18	cytokeratin 18
CNS	central nervous system
CPT	carnitin palmitoyl transferase
CRTC2	CREB-regulated transcription coactivator 2
CT	computer tomography
DNL	de novo lipogenesis
EASD	European Association for the Study of Diabetes
EASL	European Association for the Study of the Liver
EASO	European Association for the Study of Obesity
ER	endothelial reticulum
ESC	embrionic stem cells
FAO	fatty acid oxidation
FAS	fatty acid synthase
FAT/CD36	fatty acid translocase/cluster of differentiation 36
FATP	fatty acid transport protein
FGF	fibroblast growth factor
FXR	farnesoid X receptor
GLP-1	glucagon-like peptide
GPCR	G protein-coupled receptor
HFD	high fat diet
HIF	hypoxia-inducible factor
HMGS	hydroxymethyl glutaryl CoA synthase
HSC	hepatic stellate cells
IL-1	interleukin 1
IP3	inositol 1,4,5 triphosphate
iPSC	induced pluripotent stem cells
IRS	insulin receptor substrate
LDL	low density lipoprotein
MCD	methionine choline deficient
mGluR5	metobotropic glutamate receptor type 5
MP	microparticles
MRI	magnetic resonance imaging
MSG	monosodium glutamate
mTOR	mammalian target of rapamicin
mTORC1	mammalian target of rapamicin complex 1
mTOT	mitochondrial target of thiazolidinediones
MTTP	microsomal triglyceride transporter
NAFLD	nonalcoholic fatty liver disease
NASH	nonalcoholic steatohepatitis
NFkB	nuclear factor kB
NOS	nitric oxide synthase
NOS	nitric oxide
OCA	obeticholic acid
PBC	primary cholangitis
PDK1	phosphoinositide-dependent protein kinase 1
PI3K	phosphoinositide 3 kinase
PIP2	phosphatidilinositol 4,5 bisphosphate
PKB	protein kinase B
PLC	phospholipase C
PPAR	peroxisome proliferator-activated receptor
PPRE	PPAR rensponse elements
ROS	reactive oxygen species
RXR	retinoic X receptor
S6K1	S6 kinase 1
Scap	SREBP-cleavage activating protein
SCD	steroyl CoA desaturase
SEC	sinusoidal endothelial cell
SRE	sterol regulatory element
SREBP	sterol regulatory element binding protein
TSC	tuberous sclerosis complex
TZD	thiazolidinedione
UCDA	ursodeoxycholic acid
US	Ultrasonography
VLDL	very low-density lipoprotein
VOC	volatile organic compound
